# Advanced Magnetic Resonance Methods in Materials Chemistry Analysis

**DOI:** 10.3390/molecules31050795

**Published:** 2026-02-27

**Authors:** Igor Serša

**Affiliations:** 1 Institute of Anatomy, Faculty of Medicine, University of Ljubljana, 1000 Ljubljana, Slovenia; igor.sersa@ijs.si; Tel.: +386-1-477-3696; 2 Jožef Stefan Institute, 1000 Ljubljana, Slovenia

For decades, magnetic resonance has been considered one of the most insightful and versatile families of analytical techniques available to chemists, physicists, and materials scientists. From the earliest implementations of nuclear magnetic resonance (NMR) spectroscopy [1,2,3,4] and electron spin resonance (ESR) [5,6], to modern high-resolution magnetic resonance imaging (MRI) [7,8,9,10,11], these methods remain unparalleled in their ability to reveal structure, dynamics, and even functional behavior at the molecular and supramolecular scales. The present Special Issue, “Advanced Magnetic Resonance Methods in Materials Chemistry Analysis”, was designed to enrich this rich tradition of innovation by presenting state-of-the-art applications, as well as to inspire future advances based on magnetic resonance techniques.

The motivation behind this Special Issue is based on the realization that many magnetic resonance methods have exceeded their original limits. For example, techniques originally developed for the elucidation of organic structures are now being repurposed, extended, and updated to address key questions in materials chemistry. These include the characterization of novel soft matter systems, the understanding of complex dynamics in polymers and gels, the detection of quantum defects in semiconductors, and imaging processes in electrochemical systems. The apparent diversity of research problems has resulted in the dispersion of studies across a variety of specialized literature, making them more difficult to access. This Special Issue addresses this problem by gathering representative contributions under a single theme, thereby increasing their visibility and maintaining thematic coherence.

An important advantage of the magnetic resonance method is its non-destructive and highly specific insight into matter. Unlike many spectroscopic techniques that provide average or bulk-averaged signals, NMR and related methods enable extremely sensitive detection of interactions conditioned by the local chemical environment and molecular motion (contribution 1). This feature enables the precise characterization of materials, ranging from novel contrast agents (contribution 2), supramolecular ion gels where ^1^H NMR relaxometry reveals collective dynamics and translational mobility (contribution 3), to ultra-small lanthanide oxide nanoparticles that enable dual imaging contrast, one for MRI and one for X-ray imaging (contribution 4).

NMR relaxometry in high magnetic fields also enables new insights into matter. For example, ^1^H relaxivity measurements over a wide frequency range provide key quantitative frameworks for understanding paramagnetic contributions in solution and allow for refinement of models of molecular interactions and relaxation dynamics that underlie many analytical applications (contribution 5). In addition to these classical NMR fields, magnetic resonance has found a new place in materials science—not only as a biomedical tool, but also as a method for visualizing processes such as dendrite growth in battery electrodes (contribution 6). Such applications highlight how MRI can serve not only as a window into living tissues, but also as a dynamic method for visualizing electrochemical processes.

A new topic covered in this Special Issue is the role of magnetic resonance spectroscopy in the investigation of high-spin defects in semiconductors and quantum materials. Techniques such as electron paramagnetic resonance (EPR) and electron-nuclear double resonance (ENDOR) reveal the complex interplay between spin, charge, and lattice in wide-band gap systems (contribution 7). These insights form the basis for new technologies in quantum information storage, spintronics, and nanoscale sensing.

In the field of materials characterization, multidimensional NMR approaches continue to push analytical boundaries. For example, two-dimensional NMR disentangles overlapping spectral features by distributing resonance data along multiple frequency axes, enabling detailed correlation and connectivity analyses that are essential for understanding complex solids and polymers (contribution 8). Meanwhile, solid-state NMR techniques such as magic angle spinning (MAS) help to average out anisotropic interactions to obtain spectra of exceptional resolution, thereby extending the reach of NMR into solids that were once unmanageable with conventional approaches (contribution 9).

Equally valuable are review and perspective papers that map the field of magnetic resonance in materials applications. For example, reviews on the high-precision determination of interaction parameters in single crystals (contribution 9) or the use of NMR for the characterization of industrial materials such as bitumen (contribution 10) illustrate how fundamental methodological advances translate into real-world analytical power. These papers not only summarize the state of the art, but also highlight challenges—such as spectral complexity, field inhomogeneities, and sensitivity limits—that continue to challenge methodological research.

Interdisciplinary perspectives further enrich this Special Issue. Nuclear magnetic resonance has emerged as a key tool in the study of clathrate hydrates (contribution 11), crystalline water frameworks that encapsulate gas molecules, with implications for energy storage as well as carbon capture and storage. Such studies demonstrate the ability of NMR to probe structure and dynamics in confined systems—an area of increasing interest in soft matter and materials chemistry.

Although the papers collected here cover a wide range of topics, they share a common thread: each exploits the unique synergy of magnetic resonance with materials analysis. Whether through sophisticated pulse sequences, innovative imaging protocols, or hybrid analytical schemes, these works collectively illustrate how magnetic resonance continues to evolve as a fundamental science and a practical analytical platform. They remind us that advances in methodology often come not from incremental adaptations but from bold transformations of the techniques, using old tools to address new questions and vice versa.

Several exciting directions lie ahead. The integration of magnetic resonance with complementary techniques—such as X-ray methods, electron microscopy, and computational modeling—promises richer, multimodal characterizations of complex materials. Advances in hardware, including ultra-high-field magnets and cryogenic-free technologies, will improve the sensitivity and resolution of NMR. On the theoretical front, machine learning and data science approaches are poised to revolutionize spectral interpretation, enabling the automated extraction of structural and dynamical parameters from increasingly complex datasets.

Finally, the articles presented in this Special Issue illustrate the vibrancy and diversity of magnetic resonance research in materials chemistry. They represent the field’s current innovations and signal its future, where magnetic resonance methods will continue to reveal the structure, behavior, and potential applications of materials at all scales. We hope that this collection will not only serve as a valuable resource for specialists, but will also inspire researchers from a variety of disciplines to embrace and expand the frontiers of magnetic resonance science.
